# An agent-based simulation of extirpation of *Ceratitis capitata* applied to invasions in California

**DOI:** 10.1007/s10340-013-0513-y

**Published:** 2013-06-29

**Authors:** Nicholas C. Manoukis, Kevin Hoffman

**Affiliations:** 1 US Pacific Basin Agricultural Research Center, United States Department of Agriculture-Agricultural Research Service, Hilo, HI USA; 2 California Department of Food and Agriculture, Sacramento, CA USA

**Keywords:** Simulation, Model, Medfly, Quarantine, Agent-based modeling

## Abstract

We present an agent-based simulation (ABS) of *Ceratitis capitata* (“Medfly”) developed for estimating the time to extirpation of this pest in areas where quarantines and eradication treatments were immediately imposed. We use the ABS, implemented in the program MED-FOES, to study seven different outbreaks that occurred in Southern California from 2008 to 2010. Results are compared with the length of intervention and quarantine imposed by the State, based on a linear developmental model (thermal unit accumulation, or “degree-day”). MED-FOES is a useful tool for invasive species managers as it incorporates more information from the known biology of the Medfly, and includes the important feature of being demographically explicit, providing significant improvements over simple degree-day calculations. While there was general agreement between the length of quarantine by degree-day and the time to extirpation indicated by MED-FOES, the ABS suggests that the margin of safety varies among cases and that in two cases the quarantine may have been excessively long. We also examined changes in the number of individuals over time in MED-FOES and conducted a sensitivity analysis for one of the outbreaks to explore the role of various input parameters on simulation outcomes. While our implementation of the ABS in this work is motivated by *C. capitata* and takes extirpation as a postulate, the simulation is very flexible and can be used to study a variety of questions on the invasion biology of pest insects and methods proposed to manage or eradicate such species.

## Introduction

*Ceratitis capitata* (Mediterranean fruit fly, “Medfly”) is a major threat to agriculture around the world because it can infest a large variety of commercial fruit crops (Liquido et al. 1990) and is able to persist in a wide variety of habitats (Messenger 1959). Growing regions that do not have resident populations of this species established or that are part of “Medfly-free” zones may occasionally experience incursions of this species, usually detected by finds of immature stages in fruit or by adult individuals being captured in traps under intensive surveillance programs. When *C. capitata* is found in an area where it is not known to be established, there are usually quarantine, eradication, and phytosanitary measures implemented to eliminate the Medfly from the infested area (CDFA 2012). Treatments are planned to last only long enough to eliminate this insect and then are lifted to allow the movement of produce safely to market without quarantine or the undue costs of excessive treatments.

One major problem that arises when commodity quarantines and other control measures are imposed as the result of a find (or “outbreak”) in a *C. capitata* “free” area is to determine how long to maintain quarantine and eradication measures before they can be lifted. This paper describes an agent-based simulation (ABS) developed to estimate how long a small population of Medfly is likely to persist in a quarantine area post-detection while the area is managed under quarantine security and eradication procedures. Specifically, we use an ABS implementation called MED-Fly Outbreak and Eradication Simulation (MED-FOES) to study the time to extirpation (local extinction) of *C. capitata* following seven actual detections of this pest that occurred in Southern California between 2008 and 2010. This is done through detailed modeling of individual flies and their life expectations based on probability density functions of survival, developmental rate, and reproductive potential determined from the actual population studies.

The state of California maintains an active surveillance network of traps, as well as a preventative release program in the Los Angeles basin using the sterile insect technique (SIT) (Dowell et al. 2000). These programs are maintained because potential establishment of the Medfly could be expected to cause approximately 1.2 billion dollars in costs to the state economy in the first year following the determination of establishment (Siebert and Cooper 1995). In addition to SIT control methods, the eradication teams implement fruit stripping, apply bait sprays, and massively increase trapping in attempts to kill all the flies in the area (Gilbert et al. 2010). Despite these efforts, wild *C. capitata* are found in the state one or more times per year, in a seasonal pattern concentrated in the summer and fall (Carey 1991). Medfly is also periodically found in other US Mainland states such as Florida (Simberloff et al. 1997), although not as frequently as observed in Southern California.

Currently, commodity quarantines and treatment duration in California following a numerically significant Medfly find are established and maintained based on a thermal unit accumulation calculation (“degree-days”; Gilbert et al. 2010), which is the prescribed method to estimate the developmental time of the insect given ambient temperatures from the infestation location. The typical quarantine and treatment duration is calculated to be the time required for three generations of the insect to pass (“three complete life cycles”) under local temperatures.

The first detection of Medfly in California occurred in 1975, and it has reoccurred regularly since the early 1980s (Carey 1996). It has been argued that Medfly may actually be established and persist at a low population density in California, with numbers increasing periodically to just above the detection level (Carey 1991). However, this matter remains unresolved, as others have argued that the Medfly in California is an example of a “metainvasion,” consisting of multiple sequential or overlapping introductions (Davies et al. 1999). Still other researchers have maintained that Medfly is repeatedly eradicated from the state (Haymer et al. 1997) or that different situations arise in different regions (Bonizzoni et al. 2001; Gasperi et al. 2002), making the situation more complex across the entire state of California.

The question of whether Medfly is in fact established in California is beyond the scope of this paper; our ABS assumes extirpation as a precept, which in the case of Medfly in Southern California may mean localized extinction from a relatively small area. This local extinction is expected to occur under quarantine and eradication procedures as implemented by the California Department of Food and Agriculture (CDFA) and the US Department of Agriculture (CDFA 2012). Applying ABS models to this situation allows an independent assessment of the extirpation process and in addition to being a practical application of a simulation modeling approach, it is a novel application of an individual-level model. The ABS presented here can (1) be considered as an independent assessment of the current three generation degree-day calculation and (2) can be used as a method of analysis to show ways in which quarantine length might be adjusted to increase effectiveness, reduce costs, or ensure uniform likelihood of extirpation following Medfly finds in a wide area.

Computer simulations of complex systems in biology are increasingly common, driven by rapid increases in processing speed, decreases in processing cost and complexity, and the realization that simulations can aid the researcher and program manager in considering added complexity of the real world, while freeing them from limitations imposed by analytical solutions of complex mathematical processes (Huston et al. 1988). The latter is particularly true for ABS models (also called “Individual-Based,” or “Multi-Agent” models), which can be minimally defined as simulations where individuals are described as unique and autonomous, and where they may interact with each other and their environment on a local level (Railsback and Grimm 2012). Such simulations describe a system of interest “from the bottom up.” This is accomplished by implementing the model using many replicate constituent agents or entities (insects in this case) and then observing the system behaviors and dynamics that result from those entities interacting autonomously with their environment, and often with each other as they would in the real world (Bonabeau 2002). Such an approach is particularly effective when working with small populations of entities as random events can significantly affect such populations. In the present study individual *C. capitata* adults and immatures are instantiated as agents (or entities) in the simulations of pest outbreaks and allowed to live, reproduce, and die based on their interactions with realistic environmental conditions.

While the freedom to include any degree of complexity in an ABS comes at a cost (DeAngelis et al. 1994; Grimm 1999; Hales et al. 2003), the pragmatic and paradigmatic benefits of the Agent-Based approach can be significant for many different areas of biology, including those concerned with pest invasions (Macal and North 2010; Railsback and Grimm 2012). These benefits have infrequently been applied to the area of insect invasion and pest management [Vinatier et al. 2011, but see Crespo-Pérez et al. (2011) for a recent example]. An ABS can be a natural description (Fry et al. 1989) of the invasion biology of an introduced insect such as the Medfly. It is particularly useful that an ABS can include discontinuities, thresholds, large effects of stochasticity in small population, and any degree of heterogeneity required to make the simulation realistic and useful for solving practical problems. In the case of the Medfly, it enables simulation of the complete life cycle of each individual (Railsback and Grimm 2012) insect in the population, which is of central importance to the question of quarantine length in our Medfly eradication assessment. Thus, the ABS approach is extremely flexible and can be modified or expanded with more data-derived information as required to address a particular problem or to ask different questions about insect invasions and their environmental repercussions. Finally, our ABS is numerically explicit and stochastic, which means that it can produce predicted expectations and associated variance, making these calculations statistically realistic and robust for real-world problem solving.

## Materials and methods

The Medfly ABS is defined using the “Overview, Design concepts, and Details” (ODD) protocol (Grimm et al. 2006, 2010), as a standardized method for describing individual-based models. The computer program we developed using ABS procedures, “MED-FOES,” is available for free as a compiled binary with the source code and documentation, online (see http://ars.usda.gov/pwa/hilo/software or http://medfoes.sourceforge.net).

### Purpose

The purpose of MED-FOES as presented here is to estimate the time to extirpation of invasive *C. capitata* under California conditions or other related habitats where Medfly-free areas might have similar climatic patterns. Results from executions of the simulation are based on parameters and input data from real outbreaks which have been used to evaluate the length of quarantine and treatment following previous and future finds of *C. capitata* in particular exclusion areas.

### Entities, state variables, and scales

**Fig. 1:**
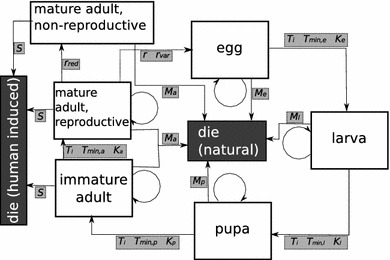
Schematic representing states and transitions possible for insects in the ABS under the simplest mode (uniform transition probability and temperature-independent mortality). The individual-level parameter *γ* is omitted for clarity

The basic entity (or agent) in our ABS is an individual insect or Medfly. These individuals are created, develop, reproduce, and die throughout the simulation in similar patterns that they would be expected to follow in nature. Figure 1 is a schematic representation of the life cycle of a single agent in the simulation, showing the stages or states in which the simulated insect can occur. During a simulation time step of 1 h, each individual insect has the potential to transition stages (develop and molt), die from natural causes, or die from human-induced mortality, in that order. If an individual dies it is removed from the simulation and no longer affects the overall calculations in the model. To further reduce the complexity and number of individual calculations, only female flies are simulated as they are the ones important in the reproduction and infestation cycle of interest to the quarantine managers.

Individual simulated insects are characterized by state variables indicating their age (in hours), developmental stage, sexual maturity, development parameters (the thermal constant *K* and base developmental temperature *T*_min_ for each stage), and a development time modifier. The individual insects (agents) are referenced to the overall “Population,” which tracks population-level data such as cumulative numbers of births and deaths. The ambient temperature for the current time step is also tracked through time, as are specific parameters relevant to each individual insect. Table 1 provides an overall summary of all the input parameters used throughout the model.

**Table 1:** Input parameters of the ABS described in this paper

Input parameter	Symbol	Units/type	Description
Temperature	*T*_*i*_	°C	Hourly temperature for time *i*
Initial population size	*N*_0_	Insects	Number of adult females at start of simulation
Daily mortality	*M*_*x*_	Proportion	Estimated daily mortality by stage
Base temperature	*T*_min,*x*_	°C	Temperature at which development rate is zero
Thermal constant	*K*_*x*_	Days	No. of day-degrees above *T*_min_ for stage transition
Maximum temperature	*T*_max_	°C	Development halts above this temperature
Developmental model	*D*_m_	Flag	Developmental transition model to use
Variation in development rate	*γ*	Proportion	SD of mean thermal units needed for stage transition
Reproductive output	*r*	Eggs	Number of eggs produced by female per reproduction
Reproductive variance	*r*_var_	Eggs	Variance of *r*
Sterilization rate	*r*_red_	Proportion	Reduction in *r* per day
Human-induced mortality	*S*	Proportion	Daily mortality from countermeasures on adults
Time to countermeasures	*t*_S_	days	Time from the first find to start human intervention
Maximum number of flies	*N*_m_	Insects	Maximum number of insects allowed in simulation

All but *T*_*i*_, *t*_S_, and *T*_max_ might be varied for the analysis of a given outbreak scenario. *D*_m_ and *N*_m_ are related to modeling and computation decisions, and are not varied between simulations of a given outbreak. *x* = e (eggs), l (larvae), p (pupae), or a (adults)

### Process overview and scheduling

As mentioned previously, our ABS functions using a 1-h time step. This level of detail is important as we consider this high resolution beneficial for overall accuracy, especially when a handful of very low or very high temperatures can have a large effect on the simulation outcome. Since hourly temperature data are available from the field, and detailed effects might be lost if daily averages were used, the simulation works to incorporate Medfly biology and development at this timescale. Thus, hourly time steps are executed in the simulation from initiation until all agents are predicted to be dead, or the maximum number of allowed steps (determined by the number of hourly temperatures available in the input file) has been met.

One hour has passed in the simulation when all the flies have been given the opportunity to age one developmental step. The following events are considered sequentially for each fly to age one step:

1. Determine if a stage transition will occur. Occurrence of a stage transition can be modeled either as a random event with the probability related only to the temperature at the current hour or as a more biologically realistic accumulation of “thermal units” with stochastic variability between individual insects. The model increments the stage and changes the developmental parameters of the individual insect if stage transition occurs to the individual entity within that time step.
2. If the insect is a mature adult and the intervention/quarantine period has not begun, one time per day the model simulates the fly laying eggs. The number of eggs produced has a mean and variance based on realistic fecundity patterns from field and laboratory data. After the start of intervention, a portion of the mature adults may become sterile each day, simulating the SIT releases. Offspring generated after intervention may optionally be sterile or subject to the same reduction in fertility as their parents.
3. Determine if the fly dies from natural causes. This is accomplished in one of two ways: (i) Natural death is a random event that occurs with a set stage-specific probability for a given simulation or (ii) stage- and temperature-specific mortality is calculated for each hour of the simulation (see “Submodels”). To enhance the realism, mode (ii) was used in all simulations applied to this study.
4. If the insect is an adult and the intervention period has begun, the model then determines if the fly dies from the intervention (human-induced death). Human-induced death is a random event that occurs with a set probability for a given simulation.

### Design concepts

#### Emergence

Individual-level variation in immature developmental rates and adult emergence, mortality, and reproductive output combined with stochastic responses to temperature lead to emergent population-level behaviors in the model. Although the Medfly population is inclined to increase based on its inherent reproductive capacity represented in the model, the initiation of countermeasures (*t*_S_) imposed by the restriction on reproduction by fruit stripping, SIT-induced reduction in fecundity and human-induced mortality imposed by bait spraying and massively increased trapping cause a general population decline as seen in the real world. It is important to note that any eggs laid after initiation of countermeasures (at *t*_S_) are considered sterile in the simulations, reflecting the expected lack of host fruit in the quarantine area following activation of countermeasures. This was implemented in the MED-FOES model to more realistically mimic the exact situation in the field; however, the generalized implementation of this ABS model allows for fertile offspring to be simulated if desired.

#### Stochasticity

At the agent level, stochasticity occurs primarily in association with developmental times, reproduction levels, and mortality rates. For developmental time, there is an individual variation in each individual’s response to ambient temperature. Reproductive output varies between adult females, with some flies being more fecund than others on an individual level. For mortality, a probability of death is calculated (see “Mortality” in Submodels section below) but the actual occurrence of death for a given individual is based on a pseudo-random number drawn each hour, and thus rates are determined both deterministically based on observed response function data and then modified stochastically to provide statistical realism in the actual mortality response within the simulation.

#### Observation

Two types of output result from a single execution of the MED-FOES model. The first is a summary file containing aggregate information like the total number of time steps (hours) that the simulation has completed, the number of alive insects throughout the simulation, and cumulative numbers of eggs produced and the deaths of adult flies. This summary also includes critical model run information (parameters, execution date, time, etc.). The second file is a detailed view of the simulation run that includes ambient temperature (min, max, and mean) and the number of living agents in each developmental stage on a daily basis. This more detailed file provides information on Medfly cohorts, especially immatures that may be incorporated in the larger habitat but unavailable to assess through the standard trapping practices.

### Initialization

In order to initiate the model, we require reasonable starting population sizes that are derived from two pieces of information based on data available in the literature and/or the monitored field situation: (1) an estimate of the size of the adult female population and (2) an estimate of the age structure of the population (especially eggs and larvae) that cannot be assessed through adult trapping. With these two estimated pieces of information, we can project the number of individuals in each life stage of the invasive population of Medfly and then initiate the model in its operational mode.

From field monitoring data provided by the California Department of Food and Agriculture (CDFA) on each of the simulated outbreaks, we have the number of flies that were found at the time of first detection. We consider flies caught in the first 3 days as the number caught at first detection. We use these values as an instantaneous measure of the adult population within the area of concern. We also know what the trapping grid was like at the time of the finds. For example, in 2008–2010: 5 Jackson traps with Trimedlure and 5 McPhail traps with protein bait were deployed per square mile (Gilbert et al. 2010). Based on release–recapture work conducted in Hawaii, it has been estimated that the attractive ability of such traps is estimated to be 0.04 % of the population to one trap per square mile per adult life span (Steiner 1967, 1969). Steiner (1967) notes, however, that he suspected movement of the flies out of the study area which would somewhat alter the assessment values. More recent works by Cunningham and Couey (1986) and Lance and Gates (1994) indicate a somewhat better sensitivity. At five traps per square mile, which is the standard density in urban parts of Southern California, the former estimates sensitivity to be 2.21 % of males per adult life span. Lance and Gates (1994) found a lower rate, around 0.6 %.

A reasonable estimate for the detection efficiency of Trimed lure is probably around 1.0–1.5 %. However, we must also consider the additional detection ability afforded by the McPhail traps. The available literature suggests that protein lures are approximately as effective as Trimed lure in attracting Medfly (Grout et al. 2011; Katsoyannos et al. 1999; Midgarden et al. 2004). For Medfly in our analysis, then, we assume a 2–3 % sensitivity of the trapping grid. Given a number of adult females derived from assuming an even sex ratio, we use the projected stable age structures from Vargas et al. (1997) at 24 °C to infer the number of individuals in immature stages that should exist in the population at large, even though we have no actual measurement of their densities. To be conservative, we ran a second set of simulations with the initial age distribution from Vargas et al. (2000) and found very similar results, thus the model does not seem to be particularly sensitive to these initial age distribution parameters.

### Input data

#### Hourly temperatures

MED-FOES simulations use hourly temperature data acquired from the California Irrigation Management Information System (CIMIS; data available online at http://wwwcimis.water.ca.gov), which maintains weather stations logging at this rate all across the state of California. These data are available for many monitoring sites via the internet and are accuracy checked for all locations. In cases where data were missing from the time periods and locations used, they were estimated from daily min–max averages from the previous 5 years using the method described below in the “Submodels” section.

#### Outbreak events

We have simulated seven outbreak events that occurred in Southern California from 2008 to 2010. The locations of the seven outbreaks and additional details are provided in Table 2. The state of California imposes quarantine on fruit entering or leaving an area of ~210 km^2^ around each find of Medfly according to a published set of rules (CDFA 2012). The standard length of each quarantine is then determined using a fixed degree-day developmental transition model (Carey 1992) based on temperatures for the location of the find. The number of degree-days is calculated to allow the time needed for three generations of Medfly to pass (CDFA 2012). This length of time has historically proven effective in avoiding recurrence of Medfly detection in an area after interventions and when completed, the quarantine and other interventions are terminated. Trapping continues in place to both assess effectiveness and monitor for subsequent reinvasions and/or population increases.

**Table 2:** Dates and locations of outbreaks analyzed with ABS plus the distance to the nearest CIMIS weather station

Outbreak	Approx address	Find	Q. end	WS	dist
Santa Monica	Warwick Ave.	10/28/09	08/29/10	99	2.4
Fallbrook	Punta de Lomas	10/29/09	07/16/10	62	22.2
Spring Valley	Leland St.	02/05/09	08/27/09	147	16.7
Imperial beach	11th St.	07/30/09	12/21/09	184	16.3
Mira Mesa	Flanders Dr.	05/20/09	11/03/09	150	2.1
Escondido	N Rose St.	09/09/09	07/26/10	153	10.0
Cajon	Lisa Terrace.	11/07/08	07/15/09	184	22.6

Dates are given in MM/DD/YY format

*Q* quarantine, *WS* ID number of the nearest CIMIS weather station, *dist* distance from CIMIS weather station to outbreak location, in kilometers

### Submodels

#### Development

Many reports on the development of pest or other insects in the literature give the mean time to transition in days or hours (*d*) for each stage as affected by a range of constant fixed temperatures (*T*). These are commonly obtained via linear regression of the developmental rate against fixed temperatures (1/*d* = *a* + *bT*) (Campbell et al. 1974), which gives a clear linear relationship for temperatures between about 16 and 30 °C for Medfly (Grout and Stoltz 2007). The commonly reported base developmental temperature *T*_min_ and developmental constant *K* (which is the time-to-transition) are related to the terms of the linear regression model above, *T*_min_ = −*a*/*b* and *K* = 1/*b*.

We implemented a developmental model based on the thermal unit accumulation approach (Fletcher 1989), where each degree above *T*_min_ for each hour counts toward a required threshold *C* (measured in the laboratory) for stage transition. We add variation to each individual fly when it is instantiated in the form of a variable *γ*, which is the standard deviation of the development time as a proportion of the development time for each stage. Thus, when

1 $$C + \gamma \le \sum\limits_{t = 0}^{i} {T_{i} - T_{\hbox{min} } } $$

from the time of insect creation (0) to the current time *i*, stage transition occurs. Note that the value of *C* is stage-specific, and constant across individuals.

#### Reproduction

Every 24 h prior to quarantine and control intervention, every adult Medfly is simulated to lay eggs. In this sense, the model only considers females. The mean number of eggs and variance in reproductive output are set by the variables *r* and *r*_var_. After human intervention is initiated reproduction is curtailed at a set daily rate, which is denoted as *r*_red_. This variable was included based on previous models of the SIT and its effects on target insect populations (Knipling 1979), simulating the fact that, over time wild females mating with sterile males keeps them from producing viable offspring. In the MED-FOES model, each mature adult fly that exists after *t*_S_ is subject to an additional loss of reproductive ability, each day with probability *r*_red_.

#### Mortality

Two methods of simulating natural mortality are included in our ABS implementation of MED-FOES. The simplest approach is to set a fixed stage-specific daily mortality rate, which we denote as *M*_*x*_. Each hour of the simulation, and for each insect at stage *x*, a random double precision floating point number between 0 and 1 is drawn and if it is lower than *M*_*x*_^1/24^ then the insect dies.

In the case of Medfly there are reliable data available on the effect of temperature on daily mortality rates, so we used the second method for simulating death. We used the stage-specific quadratic relationships from Gutierrez and Ponti (2011) and then varied the degree of mortality between runs by varying the mortality at the optimum temperature (20–25 °C). When used this way, we denote the stage-specific mortality as *M*_x_^*^, indicating that this is the mortality at optimum, and then it is subsequently affected by temperature.

Table 3 provides the range of fixed mortality rates for each stage of the Medfly life history at approximately the optimum temperatures of 20–25 °C as determined by our survey of the literature. We added temperature-dependent mortality (*μ*_*x*_, where *x* may be e, eggs; l, larvae; p, pupae or a, adults) with parameters from (Gutierrez and Ponti 2011). The equations used were

2 $$\begin{array}{*{20}c} {\mu_{\text{e,l}} = 0.00040T^{2} - 0.0145T + 0.1314} \\ {\mu_{\text{p}} = 0.00050T^{2} - 0.0207T + 0.2142} \\ {\mu_{\text{a}} = 0.00049T^{2} - 0.0187T + 0.1846} \\ \end{array} $$

**Table 3:** Estimates of simulation parameter ranges based on literature review

Input parameter	Range (min–max)	References
*N*_0_^a^	33–100	1–4
*M*_e_^*^	0.0198–0.1200	5–11
*M*_l_^*^	0.0068–0.0946	5–10
*M*_p_^*^	0.0016–0.0465	5–10, 19
*M*_a_^*^	0.0245–0.1340	7, 12, 13, 20, 21
*S*	0.005–0.050	N/A
*T*_min,e_	9.6–12.5	5–8, 11
*K*_e_	27.27–33.80	5–8
*T*_min,l_	5.0–10.8	5–8
*K*_l_	94.50–186.78	5–8
*T*_min,p_	9.1–13.8	5–7
*K*_p_	123.96–169.49	5–7
*T*_min,a_	7.9–9.9	5, 6, 13
*K*_a_	58.20–105.71	5, 6, 13
*r*	5.0–35.0^b^	1, 2, 7, 13, 14–18
*r*_red_	0.5–1.0	22, 23

Age structure (proportions) was based on Vargas et al. 1997: 0.436 (e, eggs), 0.403 (l, larvae), 0.137 (p, pupae), 0.012 [a, adults (immature)], 0.012 [(a, adults (mature)]. *γ* was set to 0.05 and *T*_max_ to 35 for all simulations

*1* Steiner 1967, *2* Steiner 1969,* 3* Cunningham and Couey 1986, *4* Lance and Gates 1994, *5* Duyck and Quilici 2002, *6* Grout and Stoltz 2007, *7* Shoukry and Hafez 1979, *8* Vargas et al. 1996, *9* Papadopoulos et al. 2002, *10* Papachristos et al. 2008, *11* Messenger and Flitters 1958, *12* Rivnay 1950, *13* Vargas et al. 1997, *14* Back and Pemberton 1918, *15* Christenson and Foote 1960, *16* Rossler 1975, *17* Kaspi et al. 2002, *18* Harris et al. 1991, *19* El Keroumi et al. 2010, *20* Diamantidis et al. 2009, *21* Carey 2011, *22* Knipling 1979, *23* Sawyer et al. 1987

^a^Number of adult females per adult fly found

^b^ *r*_var_ estimated at 3.57 based on sample of *r* values from the literature

Additional mortality of adult flies at a rate *S* occurs after human intervention at time *t*_S_. This represents mortality from human activities such as spraying with insecticides, applying bait sprays, and increasing trapping to help control the outbreak. We set the effect of human countermeasures to vary between relatively ineffective to medium efficiency: 0.5–5.0 % and applied it as additional fly deaths per day. We do not include additional mortality in stages prior to adult as there were no measures specifically targeted against these stages in California at the time of the outbreaks under study, except for fruit stripping 100 m around the location of a find.

After *t*_S_, any new flies produced may survive, but they themselves will not reproduce due to the effects of the SIT and pesticide application programs. Flies that were alive before *t*_S_ may still reproduce, though they are subject to lose that ability at a rate set by *r*_red_ (see above). These modeling decisions represent a conservative middle ground between assuming that fruit stripping is 100 % effective with no more reproduction by any flies after countermeasures are initiated, and the perspective that the population can freely reproduce and thus would never be extirpated.

#### Estimating hourly temperatures

When hourly temperature values are not available by direct measurement, they can be estimated. In our analysis we were able to obtain direct measurements for almost all the hours being simulated (see below), but when data were missing we used the method described by Campbell and Norman (1997) to obtain estimates. The method involves using two terms of a Fourier series fitted to a longer term hourly average:

3 $$\Upgamma (t) = 0.44 - 0.46\sin (\omega t + 0.9) + 0.11\sin (2\omega t + 0.9) $$

where *ω* = /12, and t is time of day in hours, with *t* = 12 at solar noon. The temperature for any time of a day *i* can be estimated as follows:

4 $$T(t) = \left\{ {\begin{array}{*{20}c} {T_{x,i - 1} \Upgamma (t) + T_{n,i} [1 - \Upgamma (t)]} & {0 < t \le 5} & {} \\ {T_{x,i} \Upgamma (t) + T_{n,i} [1 - \Upgamma (t)]} & {5 < t \le 14} & {} \\ {T_{x,i} \Upgamma (t) + T_{n,i + 1} [1 - \Upgamma (t)]} & {14 < t < 24} & {} \\ \end{array} } \right. $$

where *T*_*x*_ is the daily maximum temperature and *T*_*n*_ is the daily minimum.

#### Validation

Agent-based methods can be powerful for simulating complex systems, but require careful testing and validation through comparison against real-world data (Bonabeau 2002). To validate the behavior of a population of flies using ABS we compared MED-FOES output under simplified conditions of constant temperature, reproduction, and mortality to that from a standard Leslie matrix projection (Leslie 1945; Lewis 1942). This is a well-studied method, and we made these comparisons using actual Medfly data and comparisons with other published models. Carey (1982) utilized the reproductive, survivorship, and developmental data from Shoukry and Hafez (1979) to study Medfly demography using a Leslie matrix model. We used the same parameters as they applied to MED-FOES with the same data and compared the results, so in the current context validation is defined as testing the ABS against a known method to assess its behavior under simple conditions.

## Results

### Validation

**Fig. 2:**
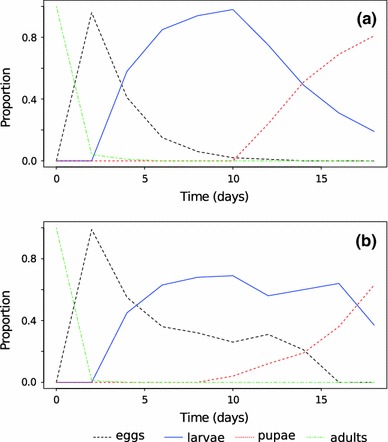
Proportion of insects in each developmental stage over 20 days under two modeling methods. **a** Leslie matrix projection and **b** ABS. Both methods used parameters measured in real insects (Shoukry and Hafez 1979), and exhibited similar demographic trends (decreasing number over time). Parameters for the Leslie matrix model were as shown in Carey (1982), with 40 age classes and the initial number of mature females *N*_30,0_ = 100. For the ABS developmental parameters were taken directly from Shoukry and Hafez (1979): *T*_min,e_ = 9.6, *T*_min,l_ = 5.0, *T*_min,p_ = 13.8, *K*_e_ = 33.4, *K*_l_ = 186.8, *K*_a_ = 124.0. Mortality rates per stage were fixed to match those used by Carey (1982): *M*_e_ = 0.00, *M*_l_ = 0.60, *M*_p_ = 0.20, *M*_a_ = 0.15. ABS-specific parameter *D*_m_ = 1 (thermal unit developmental model) was also used

Figure 2a shows a 20-day iteration of the Leslie matrix projection of Carey (1982) and Fig. 2b shows the same length of time simulated with the MED-FOES model. In both cases the population is initiated with 100 sexually mature females, allowing direct comparison. The results of the ABS are quite similar to those from the Leslie matrix approach. We note that in the case of these parameters the population size is decreasing through time under both methods, so there is increasing variance in the later days of the simulation. This is not seen in the Leslie matrix projection results (Fig. 2a) because that method is deterministic and not affected by small insect number as would be expected in the real world as densities decrease. It is noteworthy that the overall number of flies drops over time primarily as the daily survivorship of larvae derived by Carey (1982) is very low at 0.6, thus very few insects can transition to mature adults to reproduce.

**Fig. 3:**
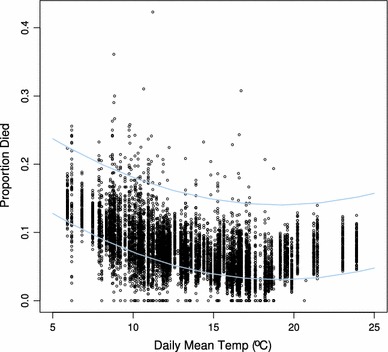
The observed relationship between mean daily temperature and the proportion of agents in all stages that died on the same day for a randomly selected subset of 100 simulations of the Fallbrook outbreak. Data points for population sizes over 25 individuals only are included. The two gray lines represent the lower and upper boundaries of daily adult mortality set for all the simulations

The behavior of populations within the ABS is determined by events occurring at the level of individual agents, subject to stochastic effects. Figure 3 shows the relationship between mean daily temperature and the proportion of agents in all stages that died during the same day for a sample of 100 executions of MED-FOES from the analysis of the outbreak in Fallbrook. It is clear that there is some relationship between the observed mortality and the expected level of adult mortality (*M*_a_), indicated by the gray lines. However, clearly there are also data points outside this range, indicating the influence of factors such as human-induced mortality, mortality of non-adults, and stochasticity.

In addition to validation using real data and comparison with Leslie matrix model projections, we also conducted tests of the basic operation of the simulation such as the degree-hour accumulation for transition, age-specific mortality, and adult fecundity. These trials yielded the results that were expected, and thus are not presented here other than to say that they increased our confidence that the constituent functions of MED-FOES were working as expected.

### Simulations of outbreaks

We executed 10,000 simulations of each outbreak listed in Table 2 using the parameters in Table 3. Parameter sets for each simulation were generated from the ranges shown in Table 3 by Latin Hypercube Sampling (Blower and Dowlatabadi 1994). Information on the times to extirpation (*t*_e_) from these simulations is given in Table 4, together with outbreak-specific parameters. In four out of seven cases the ABS showed extirpation in 95 % of the simulations within 1 month of the quarantine lengths as prescribed by the CDFA, which are known to be effective through experience. However, in three cases (Escondido, Fallbrook, and Santa Monica) the ABS gave a much shorter length of time for extirpation of 95 % of the simulations. The most extreme case, Escondido, can be explained by a cold snap in December 2009 (about 107 days after the initial find). The cold temperatures experienced in this area led to at least 4 h of temperatures below 0 °C, which greatly increases mortality in the model and thus reduced time to the predicted extirpation. These low temperatures would have the opposite effect on quarantine length as determined by degree-day analysis, as the estimated development is significantly slowed or halted by such low temperatures.

**Table 4:** Outbreak-specific parameters and results from ABS on time to extirpation

Outbreak	*N*	*t*_S_	*t*_m_	*t*_0.95_	QL	*t*_0.95_–QL
Santa Monica	4167–6250	2	160	231	297	−66
Fallbrook	2760–4167	12	143	206	273	−67
Spring Valley	1375–2083	5	136	200	204	−4
Imperial beach	1375–2083	4	123	172	145	+27
Mira Mesa	1375–2083	2	130	181	166	+15
Escondido	1375–2083	4	118	167	337	−170
Cajon	2760–4167	6	154	227	245	−18

*N* initial population size, all stages, *t*_S_ time to human intervention (days), *t*_m_ mean time to extirpation from ABS (days), *t*_0.95_ time for 95 % of the ABS runs to show extirpation (days), *QL* length of quarantine determined by 3 generations of degree-day development (days)

**Fig. 4:**
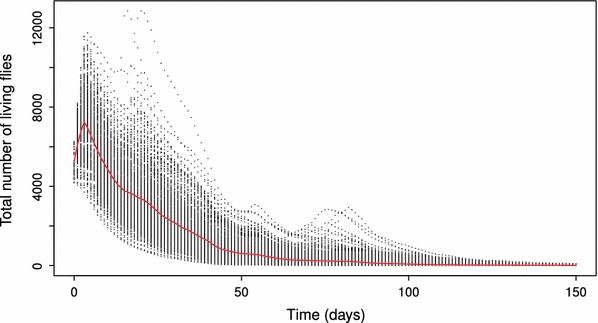
Total number of flies in 500 simulations of the Santa Monica outbreak over time. Times are truncated to be less than 150 days. The red line represents the mean number of flies across simulations

We examined the results of the ABS of the Santa Monica outbreak further. Figure 4 shows the number of flies in a subset of the simulations performed. It is clear that population size stays within the range that has been suggested to be reasonable for Medfly finds in California (Cunningham and Couey 1986): a few hundred to thousands of adults, generally. It is also clear that some simulations have multiple generations, but not all, and the number of generations and the relative size of the population in each model run are highly variable. These results indicate that the parameter ranges used for the ABS analysis of this outbreak produce a variety of possible population responses, including many realistic scenarios that may affect the actual outcome of the eradication program.

We conducted a sensitivity analysis of the ABS model for the Santa Monica outbreak using a partial rank correlation coefficient (PRCC) procedure (Kendall 1942). The PRCC test measures the strength of the association between our response variable *t*_e_ and each of the ABS input parameters while controlling for all other parameters in the model (see Table 5 for results).

**Table 5:** Sensitivity of time to extirpation for individual simulations (*t*_e_) to input parameters via partial rank correlation coefficient (PRCC) analysis

Input Parameter	Coefficient	Test statistic (*ρ*)	*p*
*M*_p_^*^	−0.60	−74.75	<0.0001
*M*_l_^*^	−0.45	−50.81	<0.0001
*r*_red_	−0.42	−45.97	<0.0001
*M*_a_^*^	−0.37	−40.21	<0.0001
*T*_min,p_	0.36	38.83	<0.0001
*r*	0.30	31.59	<0.0001
*M*_e_^*^	−0.24	−25.23	<0.0001
*S*	−0.15	−15.33	<0.0001
*K*_a_	−0.08	−8.40	<0.0001
*N*_0_	0.07	6.72	<0.0001
*K*_p_	0.06	5.89	<0.0001
*T*_min,a_	−0.06	−5.58	<0.0001
*T*_min,l_	−0.04	3.71	0.0002
*K*_l_	0.02	1.85	0.0644
*K*_e_	0.01	0.69	0.4909
*T*_min,e_	−0.01	−0.64	0.5234

Entries in the table are ordered by the magnitude of the coefficients, which indicates the relative importance of each input parameter

## Discussion

We have developed and implemented in the program MED-FOES an ABS designed to test quarantine lengths following a find of *C. capitata* in an area where it is not established. Our use of MED-FOES has yielded significant insights into population extirpation based on the actual outbreaks we simulated in this paper in two ways: (1) by comparison with the quarantine lengths set when the outbreaks occurred and (2) by analysis of the effect of simulation input parameters on its outcome. Below we discuss these two areas, place MED-FOES in the context of other insect ABS models, and conclude with expected future developments.

The predictive ability of MED-FOES for determining time to extirpation following Medfly outbreaks in California can be assessed against the length of quarantine imposed by CDFA, which is based on three generations of the deterministic degree-day development model (Table 4). The quarantine and countermeasures imposed for each of these seven outbreaks can be argued to have been successful in extirpating Medfly from the immediate area of the find (Dowell et al. 1999). This is thought to be the case as detections were not made in the same local area after quarantine and treatment; however, they may not have totally eradicated the flies from the Southern California region. Indeed the outbreak data might be credibly interpreted to suggest that there was movement of flies from a single introduction in 2008 that led to the six subsequent finds through 2010. Though this hypothesis is not the topic here, it will influence future developments of the ABS, discussed below.

Our analysis with MED-FOES provides independent verification that the quarantine lengths set at the time of the outbreaks were appropriate for extirpation in the immediate area of the finds. This was the situation in most of the cases examined, but the margin of safety in the duration of the quarantine and control actions was not the same in all cases. Specifically, in the cases of Fallbrook, Santa Monica, and Escondido, the ABS suggests that the quarantines were much longer than necessary for a reasonable certainty of extirpation. In the Imperial Beach and Mira Mesa cases, the ABS called for a slightly longer time for 95 % of the simulations to show extirpation, so the actual quarantine times implemented in these specific cases might not have yielded as high a certainty of extirpation as in the other outbreaks.

The ABS approach used in MED-FOES is a more realistic and comprehensive method of assessing and managing invasive Medfly populations following detection in an exclusion area when compared with the currently used degree-day approach. While the latter considers only the development of individuals, the ABS includes development together with demographic explicitness, mortality, and reproduction. Importantly, it also explicitly includes the countermeasures implemented by humans: SIT and bait sprays/increased trapping in the cases examined.

MED-FOES is more flexible than a degree-day calculation, and individual factors such as SIT may be excluded in cases where they do not apply, or other factors may be easily added into the simulation if other approaches are used for management. In addition, the ABS approach produced mean estimates of time to extirpation and also variances around these estimates, which the standard use of a degree-day calculation would not.

Systematic analysis of the behavior of the ABS can lead to novel insights into the changes in an invading population of *C. capitata* over time. The large difference between the quarantine lengths estimated by the degree-day approach versus the much shorter estimated time for extirpation from the ABS in the Escondido outbreak is an excellent example of how the ABS provides new insights and could perhaps save valuable resources for regulators and producers. At a regulatory level authorities can now consider cold snaps or any extreme condition as shortening the length of quarantine or even ending it, though they might have the opposite effect when using the currently standard developmental calculations of a degree-day model.

The sensitivity analysis presented in Table 5 shows that some parameters are of special importance to the simulation outcome aside from mortality for adults (*M*_a_^*^), which might be expected. In fact, the mortality at the optimum temperature for the larvae and pupae were more strongly related to *t*_e_ (time to extirpation). The relatively lower rank of *M*_a_^*^ may be due to augmented human-induced mortality (*S*) after intervention at *t*_S_. Sensitivity analysis of another agent-based model focusing on aphid population dynamics shows a similarly important role of mortality generally (Parry et al. 2006).

Reduction in reproductive agents through SIT (*r*_red_) was the third most important factor following pupal and larval base mortality. The top four parameters (mortality at optimum for pupae, larvae, and adults and *r*_red_) play a large role in determining if there will be one, two, or more generations of Medfly after a detected outbreak. It is important to further measure these input parameters in live insects during the larval and pupal stages to refine future ABS investigations of Medfly incursions. It is interesting to note that the initial number of flies as determined by *N*_0_ and the initial age distribution did not have a major effect on *t*_e_, indicating that the simulation is robust to errors in the estimate of the initial population size.

To our knowledge, agent-based approaches have not previously been applied to the question of quarantine lengths following insect invasions, though they have been used to address an array of other issues in insect biology, ranging from foraging networks and nest choice in ants (Jackson et al. 2004; Pratt et al. 2005) to landscape-level population studies (Arrignon et al. 2007; Babin-Fenske and Anand 2011; Perez and Dragicevic 2012) and disease vector dynamics (Almeida et al. 2010). The majority of the agent-based models on insects we have seen, including those cited above, are focused on adult behavior and how it scales up to population-level characteristics. The best recent models are often spatially explicit.

Though the current version of MED-FOES does not include adult behavior or explicit consideration of space, it does include a detailed developmental model, which is relatively uncommon in insect ABSs [but see Cormont et al. (2012) and Radchuk et al. (2013) for examples]. One of the important reasons that the detailed models of development are usually omitted is that a large number of developmental parameters measured in the lab or in nature are required for such a sub model to be included, and these are usually not available (Holcombe et al. 2012). This is the case even in ABS models of agricultural pests (Crespo-Pérez et al. 2011; Parry et al. 2006).

Including position and movement over a realistic landscape is an important goal for future versions of MED-FOES. Adding this factor will make it possible to address the important question of *C. capitata* establishment in California or other parts of the world. It will also be possible to test the hypothesis that a series of outbreaks may be linked by movement out of the quarantine area, leading to a find in a nearby location some time after extirpation in the original site has occurred.

MED-FOES is subject to issues common to all agent-based approaches. The most important is that to be realistic, the simulation will typically include many input parameters which need to be estimated from additional studies on real organisms. For *C. capitata* we are fortunate to have a large number of studies on development, mortality, and reproduction, but even for this species modeling movement at a landscape level will be difficult. A secondary but related problem is testing many combinations of these parameters across real-world environmental diversity, a process which can be computationally expensive. As a result of this added complexity, an ABS can often produce highly precise predictions that may not be particularly general or realistic (Levins 1966). The hourly simulation implemented in MED-FOES requires the best possible input data, specifically hourly temperature data. In some cases the nearest CIMIS weather station was over 20 km away, which might introduce inaccuracy in the simulation.

The ABS presented here might be generally considered a first draft of a framework for studying insect invasions. It can already be used to test if establishment of a given insect in a particular area is likely or unlikely, especially if sterility after human intervention is not assumed (this option is available in the current version of MED-FOES) and perhaps with extensions to consider temporal variation in habitat suitability (Arrignon et al. 2007). In the current paper we have used MED-FOES to gain insight into the time to extirpation of invasive Medfly in California focusing on the sufficiency of the length quarantine and intervention used by the state in seven outbreaks. Future development of this ABS is expected to include the implementation of males, adult behavior, and consideration of movement over the actual landscape of an outbreak and other spatially explicit elements (such as sterile flies and fruit stripping). These additional sub-models will allow for true interaction between the agents in the simulation, a common element of other ABS that can lead to emergent properties (Bedau 1997) from the system and allow a deeper exploration of insect invasions.
